# Distance from Construction Site and Risk for Coccidioidomycosis, Arizona, USA

**DOI:** 10.3201/eid2009.131588

**Published:** 2014-09

**Authors:** Janis E. Blair, Yu-Hui H. Chang, Yvette Ruiz, Stacy Duffy, Beth E. Heinrich, Douglas F. Lake

**Affiliations:** Mayo Clinic Hospital, Phoenix, Arizona, USA (J.E. Blair); Mayo Clinic, Scottsdale, Arizona (Y.-H. H. Chang, B.E. Heinrich);; Arizona State University, Phoenix (Y. Ruiz, S. Duffy, D.F. Lake)

**Keywords:** Coccidioides, coccidioidomycosis, fungal infection, fungi, Arizona, construction site

## Abstract

*Coccidioides* spp. fungi, which are present in soil in the southwestern United States, can become airborne when the soil is disrupted, and humans who inhale the spores can become infected. In 2012, our institution in Maricopa County, Arizona, USA, began a building project requiring extensive excavation of soil. One year after construction began, we compared the acquisition of coccidioidomycosis in employees working adjacent to the construction site (campus A) with that of employees working 13 miles away (campus B). Initial testing indicated prior occult coccidioidal infection in 20 (11.4%) of 176 campus A employees and in 19 (13.6%) of 140 campus B employees (p = 0.55). At the 1-year follow-up, 3 (2.5%) of 120 employees from campus A and 8 (8.9%) of 90 from campus B had flow cytometric evidence of new coccidioidal infection (p = 0.04). The rate of coccidioidal acquisition differed significantly between campuses, but was not higher on the campus with construction.

## Abstract 2

Working adjacent to a project involving excavation of desert soil did not increase the risk for infection.

The fungal infection coccidioidomycosis, which is also called Valley fever, is caused by *Coccidioides* spp. and is acquired through inhalation of airborne spores. Of the estimated 150,000 infections that occur annually, ≈60% occur in Arizona, USA. In Arizona, Maricopa County has been the center of a coccidioidal epidemic for years (*1*). Coccidioidomycosis is the second most commonly reported infectious disease in Arizona (*2*), although reported cases are likely an underestimate of the true number of cases. Respiratory illness develops in persons with symptomatic infection. The severity of illness varies from person to person: some patients require prolonged medical evaluation, time away from work or school, treatment, or hospitalization (*2**,**3*). In 2007, estimated hospital-related charges for coccidioidomycosis totaled $89 million in Arizona (*3*). It has been estimated that 3% of the population residing in *Coccidioides* spp.–endemic areas is infected annually (*4*); thus, even if up to 60% of the infected population is asymptomatic, the potential number of patients who may lose the ability to perform daily activities, work, or go to school because of illness is substantial.

Once a person is infected with coccidioidomycosis, the immune system mounts a complex reaction to control the infection; this reaction eventually results in the presence of cell-mediated and humoral immunity (*5*,*6*). The cell-mediated immunity is measured by using a delayed-type hypersensitivity (DTH) skin test (*5*) or an in vitro assay of cellular immunity to *Coccidioides* spp.

In areas of the US Southwest where *Coccidioides* spp*.* are endemic, the fungi grow in the top 18 inches of soil. Climate and soil conditions in the area foster growth of the fungi, and after rainfall, the fungi proliferate in the form of arthroconidia. As the weather dries, arthroconidia break off and become airborne spores when the soil is disrupted (*7*). Situations and activities that increase exposure to dust increase the risk for coccidioidomycosis in humans (*7*); these situations and activities include, but are not limited to, dust storms, earthquakes, construction work, outdoor occupations or activities, and military maneuvers (*7*). Little data exist to quantify the effects of construction activities on the local epidemiology of coccidioidomycosis. Measures to control construction-associated dust have been codified into law, but no data exist to demonstrate the efficacy of these mandatory, dust-control measures in eliminating airborne arthroconidia or associated coccidioidal infections.

In late 2011, our institution embarked on the construction of a new medical facility at the site of a previously undisturbed native desert area in Maricopa County (hereafter referred to as campus A). This construction project required a year-long process of excavation and hauling of large amounts of desert soil. With the current study, we sought to quantify and compare the rate of acquisition of coccidioidomycosis among employees working at an existing facility on campus A with that among employees working at another campus 13 miles away (hereafter referred to as campus B).

## Methods

After approval was given by the Mayo Clinic Institutional Review Board, all employees at the 2 campuses were invited by email to participate in the study. Employees were included if they were >18 years of age, spent >95% of their work time on a single campus (A or B), and were self-reported to be immunocompetent. Exclusion criteria included the following: presence of any immunosuppressive illness or medication (including seropositivity for HIV; history of hematologic malignancy; and receipt of cancer chemotherapy, antirejection medication, inhibitors of tumor necrosis factor, or other immunosuppressants); a history of anergy to tests of DTH, unless subsequent skin test reactivity had been demonstrated; a history of coccidioidal illness (diagnosed by a physician or confirmed by skin testing or serologic, microbiologic, or pathologic evidence); a history of positive results for coccidioidal serology or coccidioidal skin test; current use of an oral or intravenous antifungal drug (azole or amphotericin) that could prevent coccidioidomycosis; or current pregnancy (because of a theoretical decrease in cellular immunity).

During January 22–February 13, 2012, employees who provided verbal consent completed a questionnaire to ascertain whether they met inclusion criteria and to provide additional information, such as demographic information (sex, race/ethnicity, duration of residence in the *Coccidioides* spp.–endemic area, and residential zip code); the types of regular outdoor activities they participated in; and any perception they might have that construction was occurring near their area of employment or residence. A 10-mL blood sample was collected from each participant and assayed for cellular immunity to *Coccidioides* spp. All campus A participants were recruited and had a blood sample collected before excavation and construction began. Campus B participants were recruited and tested within 2 weeks of construction onset. Twelve to 13 months later, during January 29–March 27, 2013, we again collected and assayed blood samples from participants and administered a second questionnaire. Data were eliminated from analysis if a participant’s employment site changed from 1 campus to the other after enrollment.

We used a whole-blood CD69 lymphocyte-activation assay to determine whether study participants were infected with *Coccidioides* fungi; the assay methods used were similar to previously described methods (*8*–*10*). In brief, we incubated 0.5 mL of whole peripheral blood with 5 μg of coccidioidin filtrate (provided by Mitch Magee, Arizona State University, Tempe, AZ, USA) for 24 h at 37°C in a humidified incubator containing 5% CO_2_. Phytohemagglutinin lectin (5 μg) was used as a positive stimulatory control, and 10 μL of phosphate-buffered saline (PBS) was added to the control tubes. After the 24-h incubation, we lysed the erythrocytes by using BD FACS lysing solution (Becton, Dickinson and Company, Franklin Lakes, NJ, USA) according to the manufacturer’s instructions. We resuspended the resultant peripheral blood mononuclear cell (PBMC) pellet in 200 μL of PBS and then added 20 μL each of fluorescein–conjugated anti-CD3 and phycoerythrin-conjugated anti-CD69 antibodies (Becton, Dickinson and Company). The antibodies and PBMCs were gently mixed, incubated for 30 min at room temperature, and then washed twice with 3 mL of PBS. The final PBMC pellet was resuspended in 500 μL of PBS and analyzed on a Becton Dickinson CyAn flow cytometer. Before each flow cytometry run, the instrument was calibrated according to the manufacturer’s protocol. Isotype controls for fluorescein isothiocyanate–labeled and phycoerythrin-labeled CD3 and CD69 antibodies were used to establish a CD3-positive cell gate. From that CD3-positive population, we quantified CD69-positive cell populations.

For the initial assay in 2012, we classified test results for all participants into 3 groups: definite negative (mean fluorescence intensity of CD69 above control; range 0%–5.9%), possibly negative (intermediate mean fluorescence intensity; range 6.1%–8.1%), and definite positive (mean fluorescence intensity; range 9.4%–33.1%). On the basis of results from healthy controls with known or no known history of definite coccidioidomycosis, we used 6.1% as a cutoff for differentiating between study participants with a positive or a negative test result for coccidioidomycosis. For participants eligible for the second test in 2013, a similar process was undertaken.

The percentage of employees who converted from a negative to a positive test result was calculated for each study site and compared by using the χ^2^ test or the Fisher exact test, as applicable. For other employee characteristics, categorical variables were reported in numbers and percentages and compared by using the χ^2^ test or the Fisher exact test; for the ages of participants, we reported the medians and compared them by using the Wilcoxon rank sum test.

After reviewing the results of our study, we conducted a logistic regression analysis, using only information collected in the initial questionnaire, to explore possible factors associated with conversion of cellular immunity. The univariate analysis was performed first, and any variables with p<0.30 were considered in the model-selection process. We used the backward elimination procedure to identify the variables, and any variable with p<0.15 was retained in the model. Since the comparison between employees from different campuses was of interest, campus location was retained in the model. These liberal criteria were used for the exploratory purposes of our analysis. For the final model, adjusted odds ratios (ORs), 95% CIs, and p values were reported. All analyses were conducted by using SAS 9.2 (SAS Institute Inc., Cary, NC, USA). All tests were 2-sided; p<0.05 was considered statistically significant.

With regard to the construction, the medical institution’s Construction Safety and Infection Control policy required that the contractor develop a plan for using every appropriate precaution to avoid or limit dust in the air and in adjacent buildings during construction. The plan included precautions compliant with the Maricopa County Air Quality Department specifications to limit dust pollution (*11*), including a dust control permit (*11*). Trained county inspectors made unannounced inspections of the construction site.

Construction commenced in late January 2012, and most of the excavation and movement of dirt was completed within 1 year. During that time, 15 unannounced inspections were conducted, and no violations of dust control regulations were documented. In total, 154,600 cubic yards of soil was excavated to a depth of 33 feet; pockets for concrete and steel caissons were excavated another 40–90 feet. The top 18 inches of soil was removed in the first 4 months. All excavated soil was initially moved to on-site stockpiles, but from the fourth month onward, 80% of the stockpiled soil was hauled off site; the balance of soil was re-used on the construction site for backfill or for building up new parking lots or an electric substation. It is not known when the movement of topsoil was completed or how much of the original top 18 inches of soil was in the soil that was re-used.

## Results

In January 2012, campus A employees were recruited and enrolled in the study during the 2 weeks before onset of the construction site excavation; campus B employees were enrolled 2 weeks later. A total of 316 employees met inclusion criteria: 176 from campus A and 140 from campus B. Of these employees, 20 (11.4%, 95% CI 6.7%–12.1%) from campus A and 19 (13.6%, 95% CI 7.9%–19.2%) from campus B were excluded because test results for the CD69 lymphocyte–activation assay were positive, indicating previous coccidioidal infection and current immunity (p = 0.55).

**Figure 1:**
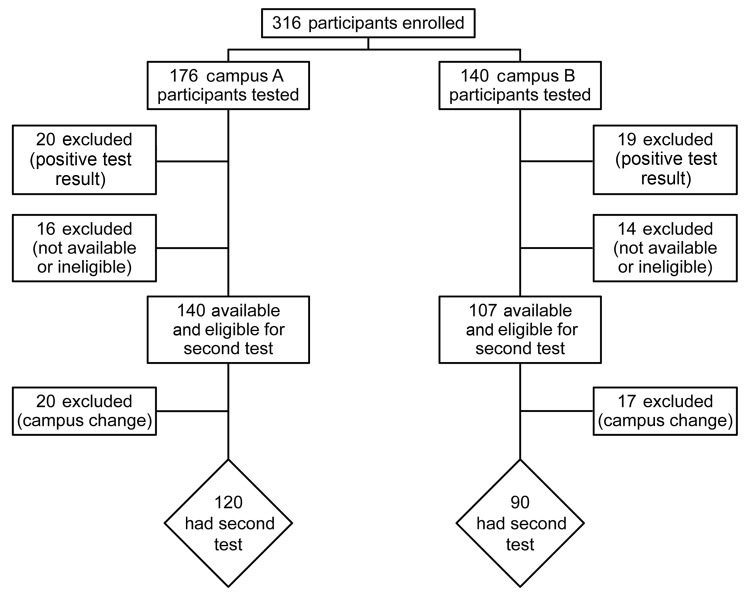
Flowchart of study participants in a study of the acquisition of immunity to *Coccidioides* spp. among persons working adjacent to and 13 miles away from a construction project requiring extensive excavation of soil, Arizona, USA, 2012–2013.

The flow of study participation, from the beginning to the end of the study, is summarized in Figure 1. After an initial positive test result, making participants ineligible for the second test a year later, the most common reasons for exclusion were employee attrition, change of employment campus, or medical leave (n = 16 for campus A; n = 14 for campus B). A year after the study was initiated, campus A had 140 eligible employees available for participation, of whom 120 (85.7%) continued in the study, and campus B had 107, of whom 90 (84.1%) continued.

**Figure 2:**
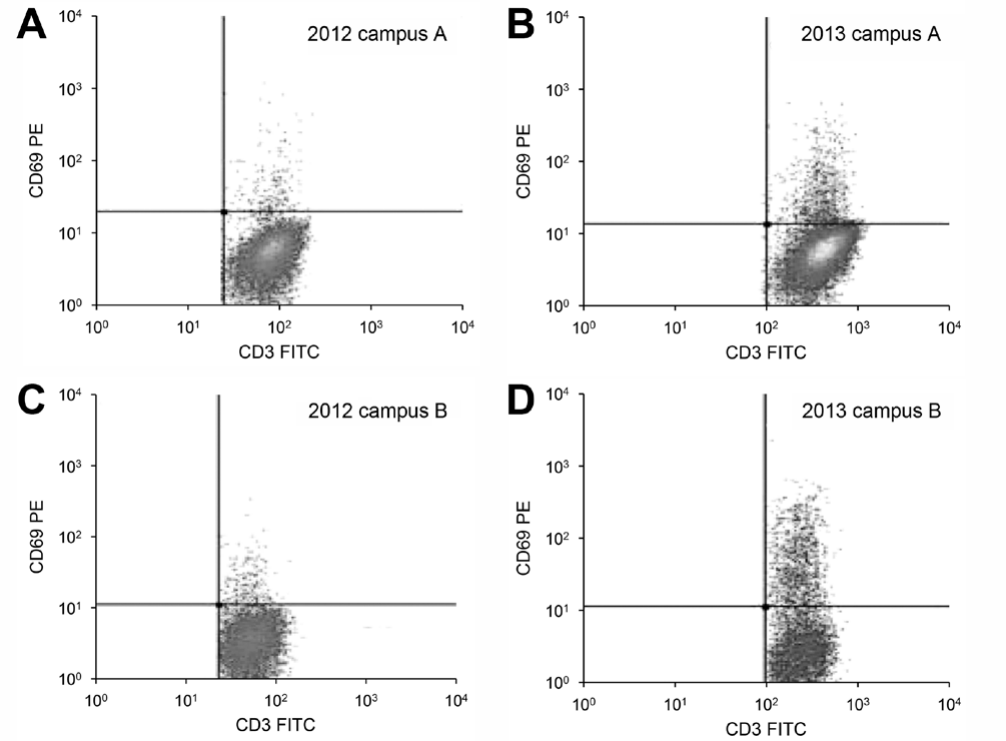
Serial flow cytometry images showing immunologic conversion from negative to positive for participants in a study of distance from a construction site as a risk factor for coccidioidomycosis, Arizona, USA, 2012–2013.Conversion was measured by using the CD69 lymphocyte-activation assay. A, B) Images for a representative participant from campus A, which was adjacent to the construction site. C, D) Images for a representative participant from campus B, which was 13 miles from the construction site. A, C) Images were done in 2012, before construction began. B, D) Images were done in 2013, a year after construction began. The participants’ CD3-positive T-cell populations are shown in the lower right quadrant of each image. The percentage of CD3/CD69-positive T cells changed from 1.9% to 6.4% in the campus A participant and from 2.9% to 17.7% in the campus B participant. FITC, fluorescein isothiocyanate; PE, phycoerythrin.

At the 1-year follow-up, 3 (2.5%) of 120 participants from campus A who had previously negative test results had lymphocyte proliferation evidence of newly acquired coccidioidal infection, compared with 8 (8.9%) of 90 participants from campus B (p = 0.04). Figure 2 shows test results for representative study participants from each campus who showed immunologic conversion from negative for coccidioidal infection in 2012 to positive in 2013.

Table 1 summarizes the demographics, perceptions of risk for coccidioidomycosis, and outdoor activities of the study population. Campus B employees were older and more likely to regularly walk outdoors than were campus A employees. At the 1-year follow-up, there was a disproportionate drop in male participants on campus B and an increase in the proportion of participants on campus B who reported construction activity near their homes. Table 2 summarizes the comparison of demographic characteristics and risk factors for coccidioidomycosis among participants who did and those who did not show immunologic conversion after 1 year. Campus location and walking outdoors for recreation were associated with conversion of cellular immunity. Participant variables, including age, participation in other (or any) outdoor activities, and residential zip code, were assessed by logistic regression, and did not correlate with conversion of cellular immunity (data not shown).

**Table 1:** Characteristics of participants, at enrollment and 1 year later, in a study of distance from a construction site as a risk factor for coccidioidomycosis, Arizona, USA, 2012–2013*

Characteristic	At enrollment, N = 316				At 1-year follow-up, N = 210		
	Campus A†	Campus B†	p value		Campus A†	Campus B†	p value
Sex							0.03‡
M	28/176 (15.9)	20/140 (14.3)	0.69‡		22/120 (18.3)	7/90 (7.8)	
F	148/176 (84.1)	120/140 (85.7)			98/120 (81.7)	83/90 (92.2)	
Median age, y (range)	47 (21–75)	53 (18–76)	0.04§		49 (23–72)	53 (25–76)	0.04§
Race							0.75‡
White	144/176 (81.8)	119/140 (85.0)	0.66‡		101/120 (84.2)	75/89 (84.3)	
Hispanic	13/176 (7.4)	10/140 (7.1)			7/120 (5.8)	7/89 (7.9)	
Other	19/176 (10.8)	11/140 (7.9)			12/120 (10.0)	7/89 (7.9)	
Indoor work location	168/176 (95.5)	137/140 (97.9)	0.35‡		117/119 (98.3)	89/90 (98.9)	0.67‡
Work near a construction site	77/168 (45.8)	2/140 (1.4)	<0.001‡		86/114 (75.4)	4/89 (4.5)	<0.001‡
Live near a construction site	18/170 (10.6)	6/135 (4.4)	0.048‡		17/120 (14.2)	8/88 (9.1)	0.27‡
New home construction, remodeling, landscaping in home or neighborhood since enrollment¶	NA	NA	NA		28/116 (24.1)	19/88 (21.6)	0.67‡
Regular weekly participation in outdoor activities#							
Running	26/176 (14.8)	23/140 (16.4)	0.69‡		18/120 (15.0)	9/90 (10.0)	0.28‡
Hiking	52/176 (29.5)	39/140 (27.9)	0.74‡		34/120 (28.3)	20/90 (22.2)	0.32‡
Walking	107/176 (60.8)	110/140 (78.6)	0.007‡		72/120 (60.0)	71/90 (78.9)	0.004‡
Yard work	53/176 (30.1)	53/140 (37.9)	0.15‡		37/120 (30.8)	33/90 (36.7)	0.37‡
Received diagnosis of coccidioidomycosis during study period	NA	NA	NA		1/117 (0.9)	0	>0.99**
Initiated antifungal therapy after enrollment	NA	NA	NA		1/120 (0.9)	1/90 (1.1)	>0.99**

*Campus A was adjacent to and campus B was 13 miles away from the construction site, which required extensive excavation of soil inhabited by *Coccidioides* fungi. NA, not available. †Values are no. with characteristic/no. total (%), except as noted for age. ‡By χ^2^ test. §By Wilcoxon rank sum test. ¶Work that took place within the past year. #Other activities that were evaluated but did not differ significantly between campuses were jogging, gardening, landscaping, golfing, playing team sports, swimming, and biking. **By Fisher exact test.

**Table 2:** Characteristics of participants, by cellular immunity conversion status at 1-year follow up, in a study of distance from a construction site as a risk factor for coccidioidomycosis, Arizona, USA, 2012–2013*

Characteristic	Cellular immunity status†		p value‡
	Negative, n = 199	Positive, n = 11	
Sex			0.65
M	27/199 (13.6)	2/11 (18.2)	
F	172/199 (86.4)	9/11 (81.8)	
Median age, y (range)	50.0 (23.0–76.0)	52.0 (26.0–71.0)	0.88
Race			0.66
White	165/198 (83.3)	11/11 (100)	
Hispanic	14/198 (7.1)	0	
Other	19/198 (9.6)	0	
Work near a construction site	87/192 (45.3)	3/11 (27.3)	0.35
Live near a construction site	23/198 (11.6)	2/10 (20.0)	0.34
New home construction, remodeling, landscaping in home or neighborhood since enrollment§	40/198 (20.2)	1/9 (11.1)	0.69
Regular weekly participation in outdoor activities¶			.
Running	26/199 (13.1)	1/11 (9.1)	>0.99
Hiking	51/199 (25.6)	3/11 (27.3)	>0.99
Walking	133/199 (66.8)	10/11 (90.9)	0.18
Yard work	64/199 (32.2)	6 /11 (54.6)	0.19
Employment site			0.06
Campus A	117/199 (58.8)	3/11 (27.3)	
Campus B	82/199 (41.2)	8/11 (72.7)	

*A CD69 lymphocyte-activation test was used to determine if the cellular immunity status of participants had converted from negative to positive. †Values are no. with characteristic/no. total (%), except as noted for age. ‡For age, the Wilcoxon rank sum test was used; for other variables, the Fisher exact test was used. §Work that took place within the past year. ¶Other activities that were evaluated but that did not differ significantly between participants who did and did not convert from negative to positive cellular immunity status were jogging, gardening, landscaping, golfing, playing team sports, swimming, and biking.

The final model, which evaluated factors associated with the conversion of cellular immunity, showed that participants on campus A, compared with those on campus B, had a lower odds of acquiring coccidioidal infection (adjusted OR 0.42, 95% CI 0.15–1.19; p = 0.10). Regularly taking walks outdoors was associated with increased odds of acquisition (adjusted OR 3.39, 95% CI 0.74–15.49; p = 0.11).

During the 1-year study period, 1 participant had a clinical episode consistent with new coccidioidal infection. This otherwise healthy 54-year-old woman experienced an insidious onset of heart palpitations, dyspnea, cough, profound fatigue, and new back pain; chest imaging showed a 12-mm solid pulmonary nodule with satellite lesions that were not present in a radiograph from 4 years earlier. The results of coccidioidal serologic testing were positive by enzyme immunoassay for IgG and indeterminate for IgM; immunodiffusion was indeterminate for IgM. This participant received a clinical diagnosis of probable coccidioidomycosis; she recovered clinically and had negative coccidioidal serology results within 6 months, without antifungal treatment. Results of her enrollment and follow-up lymphocyte activation studies were negative. During the study period, short-term (<2 weeks’ duration) antifungal treatments were administered to 2 other study participants (1 from each campus) for noncoccidioidal illnesses.

## Discussion

Coccidioidomycosis is a respiratory illness with a variety of clinical manifestations. Approximately two thirds of infected persons are asymptomatic; the remainder show signs and symptoms of systemic and respiratory illness that range from mild to severe and life threatening.

Once a person is infected with *Coccidioides* fungi, the immune system mounts a complex reaction to control the infection; this reaction eventually results in the presence of cell-mediated and humoral immunity (*5*,*6*). Persons who recover uneventfully from coccidioidomycosis become immune and are unlikely to have subsequent coccidioidal infections. Such immunity can be assessed (regardless of the presence or absence of symptomatic illness) with a DTH skin test (*5*) or an in vitro assay of cellular immunity to *Coccidioides* spp., such as the assay described in this report. Because the DTH skin test is not commercially available, we elected to use a lymphocyte activation assay to identify any study participants with such immunity (*10*). As reported by Ampel et al. (*10*,*12*), this assay detects the activation marker CD69 on the surface of CD3+ T cells, correlates well with skin test reactivity, and indicates previous (or current) exposure to *Coccidioides* spp. Although Johnson et al. (*13*) later used T27K, a coccidioidal antigen preparation, we elected to use coccidioidin filtrate, which has historically been shown to be a good coccidioidal preparation for DTH testing and to be an even better preparation for determining cellular immunity in vitro (*14*). We chose to use the lymphocyte activation assay rather than standard serologic testing to measure cellular immunity for 3 reasons: 1) serology, while often adequately sensitive for evaluation of clinical illness, is not 100% sensitive (*15*); 2) serologic sensitivity depends on the time from onset of symptoms and may be undetectable in early or resolved illness (*15*,*16*); and 3) in the absence of clinical illness, it may be difficult to distinguish true-positive from false-positive serologic testing results (*17*). Up to 60% of infections may be asymptomatic, so we wanted an assay that would measure asymptomatic infection.

*Coccidioides* fungi naturally reside in the top 18 inches of soil in areas where *Coccidioides* spp. are endemic. However, even within such areas, soil sampling studies aimed at isolating the fungus by culture or by molecular amplification have shown the distribution of *Coccidioides* fungi to be spotty and erratic, even where the fungi are highly prevalent (*18*,*19*). We did not undertake soil sampling studies before construction began, and it is certainly possible that, unbeknownst to us, the soil of the 2 campuses assessed in this study had different concentrations of *Coccidioides* fungi, and, more specifically, that the soil at the campus A construction site did not have a high level of fungal organisms.

Where present, *Coccidioides* fungi naturally reside in the top layers of soil; thus, activities that disrupt the soil and create dust, increasing the airborne dissemination of *Coccidioides* spores, are recognized as risk factors for an increased likelihood of coccidioidal acquisition. Persons engaged in construction, agriculture, archeological digs, and other soil-disrupting activities within areas where *Coccidioides* fungi are endemic have experienced increased dust exposure and subsequent coccidioidal infection (*20*–*23*). In addition, 2 reports have implicated construction as a risk for development of coccidioidomycosis among persons in the surrounding community (*24*,*25*).

In 2002, the onset of construction of a mental hospital adjacent to the Pleasant Valley State Prison in California was temporally associated with 127 new cases of coccidioidomycosis among prisoners over the subsequent 15 months; these 127 new cases compared with only 7 cases from the same institution in the preceding year (*24*). In other, more limited, observations, dust control at military bases by natural means (i.e., rainfall) or by artificial measures (e.g., planting lawns or oiling down unpaved roads and airstrips) has been associated with a temporary reduction of airborne dust and with the subsequent rate of coccidioidal infection (*25*). However, the observations in both of these reports took place over 2 sequential years, and neither study controlled for year-to-year variations in weather (e.g., rainfall, temperature, or wind) or for background cases of coccidioidomycosis within the same area.

In planning this study, we hypothesized that the dust generated from the construction on campus A would result in an increase in the acquisition of coccidioidomycosis among employees at campus A, compared with the acquisition of coccidioidomycosis among employees on campus B, 13 miles away. Knowing that dust-suppression measures would be used at the construction site, we were uncertain about what magnitude of difference to expect in infection rates. However, our findings did not show that the rate of newly acquired coccidioidomycosis was higher among study participants from campus A than among participants from campus B. In fact, the 2.5% rate of newly acquired coccidioidomycosis cases on campus A is essentially the same as the 3% rate of infection previously estimated for residents of *Coccidioides* spp.–endemic areas (*4*). Whether the construction and/or the concurrent dust control measures had any effect on the acquisition of infection is not known.

Our findings showed an overall 1-year risk of coccidioidal acquisition of 5.2% (11/210 persons; 95% CI 2.2%–8.3%) among the study participants; this rate is similar to a previous acquisition estimate of 3% per year (*4*). Rather than finding an increase of coccidioidomycosis among participants on campus A, we instead observed a statistically significantly higher rate of acquisition at the control site, campus B, which is not in an area of known higher risk for coccidioidomycosis and which had no construction being conducted on or in the vicinity of its grounds.

Several factors can affect any person’s risk for contact with arthroconidia and subsequent coccidioidal infection (e.g., recreational and other outdoor activities, exposure to dust storms, home or work location close to construction, or prevalent wind patterns). Thus, we examined demographic information provided by study participants to ascertain any risk factors among those with newly identified coccidioidomycosis. We observed an increased risk for coccidioidal acquisition not only among study participants who worked on campus B, but also a trend to significance in risk for participants at both campuses who regularly walked outdoors; no other risk factors emerged. Although walking is a common form of exercise, whether regularly walking outdoors represents a unique risk factor is not clear. In addition, this variable trended to statistical significance by virtue of a larger cohort participating in the activity; it is possible that this activity is merely an indirect marker of time spent outdoors. We also observed that the participants on each campus tended to reside in separate groups of zip codes, with only some overlap, but no particular residential zip codes were associated with a higher likelihood of infection (data not shown).

This study has several limitations. The study participants were predominantly female and white, reflecting the employee population on the 2 campuses, a factor that may limit the generalizability of our findings. In addition, the CD69 lymphocyte-activation assay has been shown to correlate with helper T cell, subtype 1 (T_h_1) cytokines, but not with T_h_2 cytokines. Therefore, if any participants had coccidioidomycosis that did not resolve because of a T_h_2 immune response, we may not have been able to detect the infection because of inadequate lymphocyte activation (i.e., a false-negative test result) (*9*). This scenario may explain the situation of the employee from campus A who had protracted, probable, symptomatic coccidioidal infection and an atypical serologic pattern, but who had a negative test result on the second CD69 lymphocyte-activation assay. Alternatively, since CD69 is a nonspecific marker of lymphocyte activation, an immune response to another infectious agent would have elevated a participant’s baseline CD69 level, making it difficult to determine whether their PBMCs were responding to coccidioidin or another infection or both.

In summary, by using the CD69 lymphocyte-activation assay, we determined that employees working adjacent to a large construction project involving the excavation of previously undisturbed native desert soil and the use of active dust-control measures, compared with co-workers at another site 13 miles away, did not have an increased risk for acquisition of coccidioidomycosis. That the control group of employees on the second campus had a statistically higher rate of negative to positive assay conversion at 1 year is a finding that merits further study.
